# The Protective Effect of Ubiquinone against the Amyloid Peptide in Endothelial Cells Is Isoprenoid Chain Length-Dependent

**DOI:** 10.3390/antiox10111806

**Published:** 2021-11-13

**Authors:** Javier Frontiñán-Rubio, Yoana Rabanal-Ruiz, Mario Durán-Prado, Francisco Javier Alcain

**Affiliations:** 1 Department of Medical Sciences, Faculty of Medicine, University of Castilla-La Mancha, 13071 Ciudad Real, Spain; Javier.Frontinan@uclm.es (J.F.-R.); Yoana.Rabanal@uclm.es (Y.R.-R.); 2 Oxidative Stress and Neurodegeneration Group, Regional Centre for Biomedical Research, University of Castilla-la Mancha, 13071 Ciudad Real, Spain

**Keywords:** Alzheimer´s disease, coenzyme Q, ubiquinol, NADPH oxidase, amyloid beta, endothelial cells

## Abstract

Vascular brain pathology constitutes a common feature in neurodegenerative diseases that could underlie their development. Indeed, vascular dysfunction acts synergistically with neurodegenerative changes to exacerbate the cognitive impairment found in Alzheimer’s disease. Different injuries such as hypertension, high glucose, atherosclerosis associated with oxidized low-density lipoprotein or inflammation induce NADPH oxidase activation, overproduction of reactive oxygen species, and apoptosis in endothelial cells. Since it has been shown that pretreatment of cultured endothelial cells with the lipophilic antioxidant coenzyme Q10 (CoQ10) displays a protective effect against the deleterious injuries caused by different agents, this study explores the cytoprotective role of different CoQs homologues against Aβ_25–35_-induced damage and demonstrates that only pretreatment with CoQ10 protects endothelial brain cells from Aβ_25–35_-induced damage. Herein, we show that CoQ10 constitutes the most effective ubiquinone in preventing NADPH oxidase activity and reducing both reactive oxygen species generation and the increase in free cytosolic Ca^2+^ induced by Aβ_25–35_, ultimately preventing apoptosis and necrosis. The specific cytoprotective effect of CoQ with a side chain of 10 isoprenoid units could be explained by the fact that CoQ10 is the only ubiquinone that significantly reduces the entry of Aβ_25–35_ into the mitochondria.

## 1. Introduction

Alzheimer’s disease (AD) is characterized by the presence of senile plaques of beta-amyloid and neurofibrillary tangles of hyperphosphorylated tau protein. The amyloid cascade hypothesis synthesizes the histopathological and genetic information and constitutes the central hypothesis that explains the onset and progression of the disease [1]. However, most patients who have received a diagnosis of sporadic AD also exhibit cerebrovascular disease [2]. Indeed, vascular brain pathology is associated with the rate of cognitive decline [3]. Longitudinal studies have suggested that high blood pressure in midlife is the most substantial vascular risk factor for both AD and vascular dementia (VaD) in later life [4], and it constitutes a major cause for the dysregulation of sustained blood flow [2], which increases the expression of hypoxia-inducible factor 1-α leading to upregulation of the beta-secretase (BACE)1 [5,6]. The generation of oxidative stress in hypertension is well defined and remains a crucial modulator of endothelial function and nitric oxide (NO) bioavailability [7]. In addition to hypertension, other risk factors for AD that increase oxidative stress are high glucose, atherosclerosis associated with oxidized low-density lipoprotein (ox-LDL), and inflammation [2]. In endothelial cells in vitro, these injuries induce overproduction of reactive oxygen species (ROS), apoptosis, NADPH oxidase activation, and the expression of cell molecule adhesion to monocytes. The pretreatment of cultured endothelial cells with the lipophilic antioxidant coenzyme Q10 (CoQ10) protects against the deleterious injuries caused by different agents [8,9,10,11]. Furthermore, endothelial cells are more sensitive than neurons to Aβ and its active fragment Aβ_25–35_, which triggers a prominent pro-apoptotic and necrotic response in these cells through mechanisms that involve an increase in free cytosolic calcium concentration ([Ca^2+^]_i_) and ROS such as O_2_^−^ and H_2_O_2_ [12,13,14,15,16]. Our previous study demonstrated that CoQ10 protects endothelial cells in vitro from Aβ-induced injury [17]. Indeed, we have recently shown that long-term supplementation with ubiquinol, the reduced form of CoQ10, can modulate the cerebrovascular response to hypoxia and prevent BACE1 expression and Aβ deposition while reducing the thickness of the cerebrovascular basement membrane (CVBM) in the 3xTg-AD mice model of AD [18]. Ubiquinol supplementation also improved cerebral vasoreactivity and reduced chronic inflammation in patients with mild cognitive impairment [19].

CoQ is produced in all cells, hence its name ubiquinone, and the major function of CoQ10 in cells occurs in the mitochondrial inner membrane, although it is also present in various amounts in different cellular membranes and LDL [20]. The CoQ molecule contains a benzoquinone ring condensed to an isoprenoid side chain synthesized through the mevalonate pathway. The side chain comprises 10 subunits in humans and *Schizosaccharomyces pombe* (CoQ10), nine in rodents (CoQ9), eight in *Escherichia coli* (CoQ8), and six (CoQ6) in *Saccharomyces cerevisiae*. In most species, one chain length of CoQ dominates, and to a minor extent, a lipid with a shorter or a longer side chain is also found, but the effectiveness as an antioxidant is not influenced by the length of the side chain [21,22]. Based on an earlier study [17], we hypothesized that the protective role of CoQ10 from Aβ-induced injury in endothelial cells could be partially due to the shielding of the membrane, which would difficult the insertion of the peptide and its further endocytosis, sorting, and trafficking to the organelles. In the present study, we demonstrate that only CoQ10 significantly reduces the entrance of Aβ_25–35_ into the cell and its sorting to the mitochondria. This is accompanied by protection from apoptosis, necrosis, and activation of NADPH oxidase. CoQ10 is also the most effective ubiquinone in reducing ROS generation and increasing Ca^2+^_i_ in endothelial bEnd.3 cells. This specific effect of CoQ with a side chain of 10 isoprenoid units seems to be a consequence of the fact that CoQ10 is the only one that significantly reduces the entrance of Aβ_25–35_ into the mitochondria in bEnd.3 cells together with the inhibition of the NADPH oxidase activation.

## 2. Materials and Methods

### 2.1. Reagents

Coenzyme Q2 and Q6 were purchased from Sigma Aldrich (#C8081, #C9504). Coenzyme Q9 was purchased from Cayman Chemical (#16866). Coenzyme Q10 was generously provided by Kaneka Corporation. Human Aβ_25–35_ (#AS-24227) and Human Aβ_25–35_ HiLyte™ Fluor 488-labeled (#AS-63308) were obtained from Anaspec. The fluorescent probes, Fluo-4 AM (#F23917), H_2_DCF-DA (#C6827), MitoSOX (#M36008), MitoTracker Red CMXRos (#M7512), Calcein-AM (#C34852), and Hoescht 33258 (#861405), were obtained from Thermo Fisher. Ethidium bromide (EtBr, #46067) was acquired from Sigma–Aldrich.

### 2.2. Aβ Preparation

Aβ_25–35_ and Aβ_25–35_ HiLyte™ Fluor 488 were prepared following the manufacturer’s instructions. To obtain Aβ fibrils, the tubes containing monomers were incubated at 37 °C for three days in a water bath. Then, the different vials were frozen at −20 °C until use.

### 2.3. Cell Culture

Brain endothelial cells derived from mouse, bEnd.3 (ATCC, CRL-2299), were maintained in Dulbecco’s Modified Eagle’s Medium (DMEM; Sigma–Aldrich, #D5796) supplemented with 10% fetal bovine serum (FBS) (Sigma–Aldrich, #F4135) and 1% antibiotic- antimycotic solution (Sigma–Aldrich, #A5955) at 37 °C and 5% CO_2_.

### 2.4. Determination of Apoptosis and Necrosis

Necrosis and apoptosis were determined as previously reported [17]. Briefly, bEnd.3 cells were seeded in 96-well plates (10,000 cells/well) and pre-incubated for 24 h with vehicle (EtOH; controls), CoQ2, CoQ6, CoQ9, or CoQ10 (5 µM). Then, the medium was removed, and cells were incubated with Aβ_25–35_ (5 µM) in fresh medium for 24 h. Finally, cells were incubated with 1 µM Calcein-AM and 10 µg/mL EtBr. Viable Calcein^+^ cells and necrotic EtBr^+^ cells were determined using fluorescence microscopy (Cytation 5, 20× objective; Biotek, Winooski, VT, USA). After image acquisition, cells were fixed and permeabilized for 4 min in ice-cold methanol, washed twice with PBS, and stained with 1 µg/mL Hoescht 33258. Apoptotic cells were determined according to morphological criteria using fluorescence microscopy (Cytation 5 multi-imaging system, 20× objective: Biotek). A total of four images/well were taken at a 20× magnification, and at least 100 cells/well were analyzed with ImageJ. The results are presented as a percentage of apoptotic or necrotic cells vs. total (*n* = 3).

### 2.5. NADPH Oxidase Quantification

NADPH oxidase was measured in the cultured media using an NADPH oxidase ELISA kit (#MBS2885477; MyBioSource, San Diego, CA, USA) following the manufacturer’s instructions. Briefly, bEnd.3 cells were seeded in 24-well plates (50,000 cells/well), pretreated for 24 h with vehicle (EtOH; controls), CoQ2, CoQ6, CoQ9, or CoQ10 (5 µM) and treated with Aβ_25–35_ (5 µM) for 24 h. Then, the culture medium was collected for the assay. The medium from the samples, the standard, and the blank sample were added to the plate covered with an anti-NOX1 antibody. After the treatment with the different reagents, diluents, and solutions, each well’s optical density (OD) was measured at 450 nm. Results are expressed as pg/10^5^ cells compared with the standard provided (*n* = 3).

### 2.6. Determination of O_2_^−^, H_2_O_2,_ and Free Cytosolic Ca^2+^

The levels of mitochondrial O_2_^−^, total H_2_O_2,_ and free cytosolic Ca^2+^ were measured as reported before [17]. Briefly, bEnd.3 cells were seeded in 96-well plates (10,000 cells/well) and pre-incubated for 24 h with vehicle (EtOH; controls), CoQ2, CoQ6, CoQ9, or CoQ10 (5 µM). Then, the medium was removed, and cells were incubated with Aβ_25–35_ (5 µM) in fresh medium for 24 h. For the determination of O_2_^−^ and H_2_O_2,_ the cells were incubated for 30 min with the probes MitoSOX (1 µM) and H2DCF-DA (2.5 µM), respectively. For free cytosolic Ca^2+^ quantification, the cells were incubated 15 min with the probe Fluo-4 AM (1 µM). Following PBS wash, images were acquired on live cells using a Cytation 5 multi-imaging system (20× objective). The percentage of mean intensity (relative fluorescent units, RFUs) vs. the control was determined using ImageJ 1.53 (*n* = 3; >50 cells/well).

### 2.7. Mitochondrial Status Characterization

Mitochondrial status was assessed using the Mitotracker CMXRos probe (Thermo-Fisher), a dye dependent on the mitochondrial membrane potential of living cells. bEnd.3 cells were seeded in 96-well plates (10,000 cells/well) and pre-incubated for 24 h with vehicle (EtOH; controls), CoQ2, CoQ6, CoQ9, or CoQ10 (5 µM). After medium removal, cells were treated with 5 µM Aβ_25–35_ peptide in fresh medium for 24 h. Then, cells were stained with Mitotracker CMXRos (200 nM) for 25 min, washed twice, and imaged using a Cytation 5 multi-imaging system (20× objective). The levels of mitochondrial fluorescence were assessed by Image. Results showed the levels of Mitotracker vs. control cells (*n* = 3; >50 cells/well).

### 2.8. Determination of Cellular Energy Phenotype

To analyze the cellular energy phenotype (OCR and ECAR), we used a Seahorse XFp Analyzer (Seahorse Biosciences, Agilent, Santa Clara, CA, USA), following the protocol previously set up by Divakaruni et al. [23]. bEnd.3 cells were seeded in XFp miniplates (7500 cells/well), pretreated for 24 h with vehicle (EtOH; controls), CoQ2, CoQ6, CoQ9, or CoQ10 (5 µM) and, after that, treated with Aβ_25–35_ (5 µM) for 24 h. Then, cells were incubated in Seahorse XFp base medium without phenol red (103, 193–100 Seahorse Biosciences) for 60 min at 37 °C before loading into the Seahorse analyzer. Three basal OCR and ECAR measurements were obtained within the first 20 min. Then, cells were stressed with different mitochondrial inhibitors: oligomycin (1 μM), which inhibits ATP production by the mitochondria, and carbonyl cyanide-p -trifluoromethoxyphenylhydrazone (FCCP) (0.3 μM), which depolarizes the mitochondrial membrane. To analyze the data, we used the Seahorse XFp software Wave 2.6 (Seahorse Biosciences, Agilent, Santa Clara, CA, USA). The OCR levels are represented as pmol of oxygen per minute, and ECAR levels are represented as the rate of change in mpH per minute. Data are presented as the mean ± SEM for each time point normalized to the number of cells per well (*n* = 3) as previously described [24].

### 2.9. Determination of Cellular Mitochondrial Respiration

We used the Seahorse Mito Stress test to analyze mitochondrial respiration, following the protocol previously described [23]. Briefly, bEnd.3 cells were seeded in XFp > miniplates (7500 cells/well), pretreated for 24 h with vehicle (EtOH; controls) or CoQ10 (5 µM), and treated with Aβ_25–35_ (5 µM) for 24 h. Then, cells were incubated in Seahorse XFp > base medium without phenol red (103, 193–100 Seahorse Biosciences) for 60 min at 37 °C before loading into the Seahorse analyzer. Three basal OCR measurements were obtained within the first 20 min. Then, different mitochondrial inhibitors were added [oligomycin, 1 μM; FCCP, 0.3 μM; antimycin A and rotenone, 1 μM]. The inhibitors led us to measure ATP-linked respiration, maximal respiration, and non-mitochondrial respiration, respectively. After different injections, three OCR values were automatically measured by the Seahorse XFp > software Wave 2.6. The OCR levels are represented as pmol of oxygen per minute. Data are presented as the mean ± SEM for each time point normalized to the number of cells per well (*n* = 3) as previously described [24].

### 2.10. Aβ_25–35_ Internalization and Mitochondrial Colocalization in bEnd.3 Cells

The study of Aβ_25–35_ incorporation was performed by confocal microscopy (Zeiss, LSM 880. 20× objective; Oberkochen, Germany) of living cells. bEnd.3 cells were seeded in 96-well Eppendorf Cell Imaging Plates (Eppendorf, 0030741030), pretreated for 24 h with vehicle (EtOH; controls), CoQ2, CoQ6, CoQ9, or CoQ10 (5 µM), and loaded for 30 min with Mitotracker CMXRos (1 µM) and Hoescht 33258 (1 µg/mL) for 15 min. All fluorescence parameters were adjusted using a positive control treated with Aβ_25–35_ HiLyte™ 488 for the previous 24 h as a reference. Aβ_25–35_ HiLyte™ 488 (5 µM) was added to the different wells, and images were acquired semi-automatically every two hours using confocal microscopy. The mean fluorescence of each cell was analyzed with ImageJ. Data are presented as the mean ± SEM for each time point and condition (2 wells; >30 cells/well). Aβ_25–35_ HiLyte ™ 488 mitochondrial internalization was measured at 24 h by confocal microscopy (Zeiss, LSM 880. 20× objective), using a 63× objective (Zeiss, LSM 880), and the same protocol was followed for the acquisition of images. The percentage of mitochondrial Aβ was calculated with the “JACoP” plug-in of ImageJ (*n* = 3).

### 2.11. Statistical Analysis

Data are expressed as the mean ± S.E.M. Statistical analyses were carried out with GraphPad Prism 8.0.2 (San Diego, CA, USA), using Student’s *t*-test and one-way ANOVA followed by Dunnett’s multiple comparison test. Two-way ANOVA (Dunnett’s multiple comparison test) was used for the real-time internalization studies. Significant differences were considered at * *p* < 0.05; ** *p* < 0.01; *** *p* < 0.001.

## 3. Results

### 3.1. CoQ10 but Not Shorter Side Chain CoQ Prevents Aβ_25–35_-Induced Endothelial Cell Death

**Figure 1:**
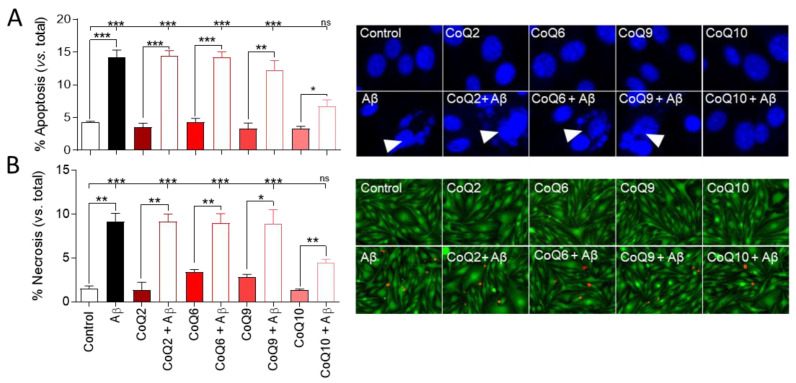
Analysis of apoptosis and necrosis in bEnd.3 cells exposed to Aβ_25–35_ and different coenzyme Q homologues (CoQs). Cells were pretreated with CoQ2, CoQ6, CoQ9, or CoQ10 for 24 h and then treated with Aβ_25–35_ for 24 h_._ (**A**) Apoptosis was determined by morphological criteria in cells stained with Hoescht 33258 (white arrows point to apoptotic nuclei). (**B**) Necrosis was determined by quantifying necrotic cells (EtBr positive cells—red) versus viable cells (Calcein-AM positive cells—green). Results show the percentage of apoptotic or necrotic cells versus total cells. Data are presented as the mean ± SEM. Student’s *t*-test was used to compare the differences between groups treated with the same ubiquinone and Dunnett’s multiple comparison test to compare each Aβ_25–35_ (plus or minus ubiquinone) treatment with the control. ns: not significant, * *p* < 0.05, ** *p* < 0.01, *** *p* < 0.001, (*n* = 3).

It was previously reported that cellular exposure to the amyloid fragment Aβ_25–35_ leads to endothelial cell toxicity by inducing cell apoptosis and necrosis [15,17], and our previous study demonstrated the cytoprotective role of CoQ10 in Aβ_25–35_-induced endothelial cell death [17]. Thereby, we explored the possible cytoprotective role of different CoQ homologues. First, we observed that the addition of those CoQ homologues (CoQ2, CoQ6, CoQ9, and CoQ10) for 24 h did not alter basal apoptotic and necrotic indexes in bEnd.3 cells, which were 4.3% and 1.5%, respectively (Figure 1A,B). As expected, adding 5 µM Aβ_25–35_ for 24 h increased the percentage of apoptotic nuclei up to 14.2% (Figure 1A), and the number of necrotic cells increased in a greater proportion from 1.5% to 9.1% (Figure 1B).

Pretreatment with different CoQ homologues (CoQ2, CoQ6, CoQ9) did not ameliorate the Aβ_25–35_-induced apoptosis and basal necrosis of bEnd.3 cells (*p* < 0.001). However, in cells pretreated with CoQ10, the percentage of Aβ_25–35_-induced apoptotic and necrotic cells was significantly lower than with other ubiquinones, and, although the number of apoptotic nuclei and necrotic cells was significantly higher than that observed in the absence of Aβ_25–35_ (*p* < 0.05), CoQ10 pretreatment was able to prevent the Aβ_25–35_-induced increase in apoptotic cells compared to the control (Figure 1A,B).

### 3.2. CoQ10 but Not Shorter Side Chain CoQ Prevents Aβ_25–35_-Induced Activation of NADPH Oxidase

**Figure 2:**
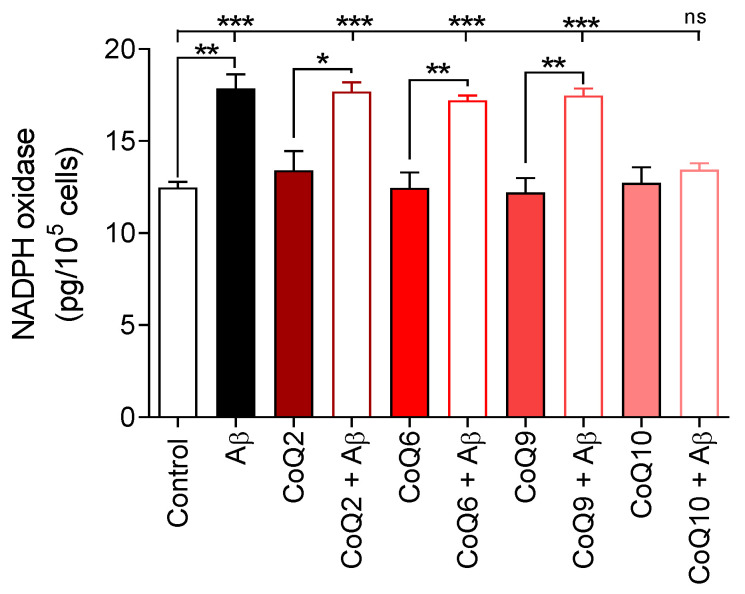
Characterization of NADPH oxidase levels. bEnd.3 cells were pretreated with CoQ2, CoQ6, CoQ9, or CoQ10 for 24 h and then treated with Aβ_25–35_ for 24 h. NADPH oxidase was assessed using a NOX1 ELISA kit. Results are expressed as pg/10^5^ cells compared with the standard. Data are presented as the mean ± SEM. Student’s *t*-test was used to compare the differences between groups treated with the same ubiquinone and Dunnett’s multiple comparison test to compare each Aβ_25–35_ (plus or minus ubiquinone) treatment with the control. ns: not significant, * *p* < 0.05, ** *p* < 0.01, *** *p* < 0.001 (*n* = 3).

Next, we investigated whether the different CoQ homologues could hamper Aβ_25–35_-induced activation of NADPH oxidase. As shown in Figure 2, basal NADPH oxidase levels in bEnd.3 cells (19.4 ± 0.86 pg/mL) were not modified after incubation with the different CoQs (CoQ2, CoQ6, CoQ9, and CoQ10).

The addition of Aβ_25–35_ (5 µM) to the culture medium significantly stimulated the NADPH oxidase levels until values of 17.85 ± 0.55 pg/10^5^ cells (*p* < 0.01) were reached and, similarly to apoptosis and necrosis, the addition of CoQ homologues with chains of 2, 6, and 9 isoprenes did not impede the Aβ_25–35_-induced activation of NADPH oxidase (Figure 2). Only preincubation with CoQ10 protected bEnd.3 cells by abolishing the increase in Aβ_25–35_-induced NADPH oxidase levels (12.71 ± 1.48 pg/10^5^ cells for CoQ10 and 13.44 ± 0.59 pg/10^5^ cells for Aβ_25–35_ plus CoQ10; Student´s *t*-test and Dunnett´s multiple comparison test).

### 3.3. CoQ10 Prevents the Aβ_25–35_-Mediated Increase in O_2_^−^, H_2_O_2,_ and Free Cytosolic Ca^2+^ Levels in Endothelial Cells and Mitochondrial Calcium Depletion in Endothelial Cells

Then, ROS production was measured by fluorescence microscopy by quantifying the levels of mitochondrial superoxide anion (O_2_^−^) and intracellular H_2_O_2,_ which were detected with the MitoSOX and H_2_DCF-DA probes, respectively.

**Figure 3:**
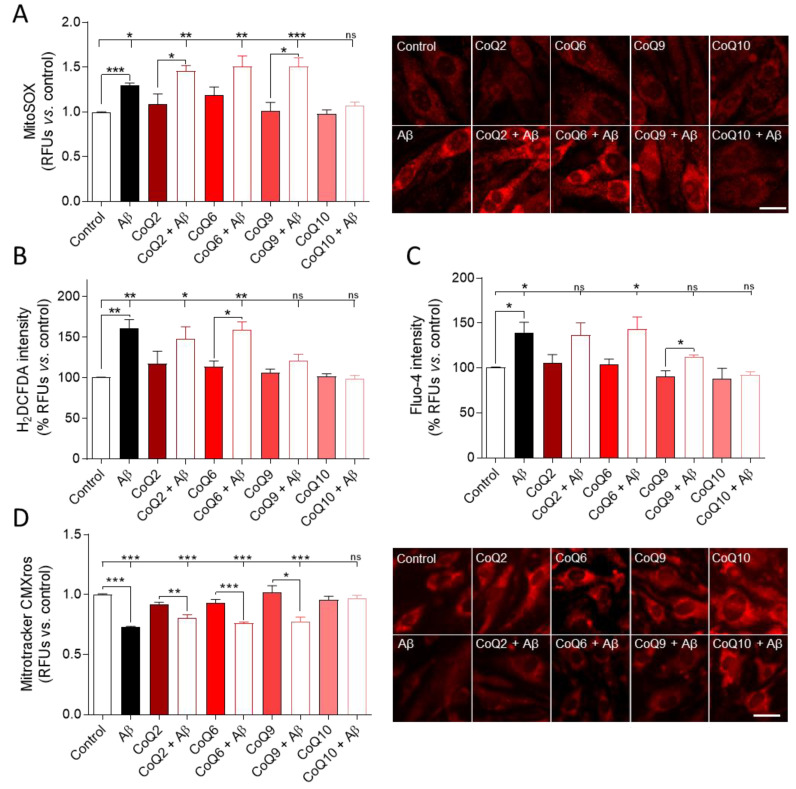
Determination of ROS levels, free cytosolic Ca^2+,^ and mitochondrial status in cells exposed to Aβ_25–35_ and different CoQs. bEnd.3 cells were pretreated for 24 h with CoQ2, CoQ6, CoQ9, or CoQ10 and then treated with Aβ_25–35_ for 24 h. (**A**) Levels of O_2_^−^ were assessed by a MitoSOX red fluorescent probe. (**B**) H_2_O_2_ levels were quantified using a H_2_DCF-DA green fluorescent probe. (**C**) Free cytosolic calcium levels were quantified using a Fluo-4 green fluorescent probe. (**D**) Mitochondrial status was assessed using a Mitotracker CMXRos probe, which is dependent on mitochondrial membrane potential. Results show the RFUs normalized vs. control cells. Data are presented as the means ± SEM. Student’s *t*-test was used to compare the differences between groups treated with the same ubiquinone and Dunnett’s multiple comparison test to compare each Aβ_25–35_ (plus or minus ubiquinone) treatment with the control. ns: not significant, * *p* < 0.05, ** *p* < 0.01, *** *p* < 0.001 (*n* = 3) (scale bar = 20 µM).

As shown in Figure 3A, bEnd.3 cells treated with Aβ_25–35_ (5 µM) for 24 h showed an increase in O_2_^−^ levels of 30% vs. untreated control cells. Treatment with the different CoQs in the absence of Aβ_25–35_ did not induce significant changes in O_2_^−^, although CoQ6 generated a slight increase. When cells were treated with 5 µM Aβ_25–35_, only CoQ10 pretreatment prevented the increase in O_2_^−^ levels, showing similar levels compared to CoQ10-treated cells in the absence of Aβ_25–35_, but this preventive effect was not observed in cells treated with CoQ2 and CoQ9. In cells pretreated with CoQ6, although Aβ_25–35_ also increased O_2_^−^ levels, the difference was not significant (Student’s test *p* < 0.015). Nonetheless, only the group > pretreated with CoQ10 showed O_2_^−^ levels with values similar to the control cells (0.97 ± 0.006 in the control vs. 1.072 ± 0.067 in CoQ10+ Aβ_25–35_; Figure 3A).

On the other hand, administration of 5 µM Aβ_25–35_ increased H_2_O_2_ levels by 60% vs. untreated controls. Incubation with some CoQs homologues slightly increased H_2_O_2_ levels, although there were no significant differences (Figure 3B). Most of them prevented the increase in H_2_O_2_ levels induced by Aβ_25–35_, thus reflecting their antioxidant activity. However, H_2_O_2_ levels observed in Aβ_25–35_ plus CoQ2 or CoQ6 were significantly higher than those found in control, and only cells cultured with Aβ_25–35_ and pretreated with CoQ9 or CoQ10 had similar levels to the control (Dunnett´s multiple comparison test, Figure 3B).

We previously reported that, in HUVECs cells, Aβ_25–35_ increased ROS levels and free cytosolic calcium [17]. This study shows a similar trend for bEnd.3 cells since Aβ_25–35_ also increased free cytosolic calcium in the absence of CoQ homologues. In the presence of CoQ2, CoQ6, and CoQ9, the free cytosolic calcium slightly increased, although there were generally no statistically significant differences. CoQ10 was the only ubiquinone that exhibited a fluorescence intensity similar to that in the control in the presence of Aβ_25–35_ (Figure 3C).

The increase in Aβ-associated oxidative stress in AD is closely related to alterations at the mitochondrial level [25]. To discern the potential protective effect of CoQs on mitochondria, we first assessed mitochondrial status by measuring the levels of MitoTracker Red CMXRos, a red fluorescent dye that is concentrated inside mitochondria by their negative mitochondrial membrane potential (MMP). Aβ_25–35_ addition resulted in a 27% reduction in MMP in bEnd.3 cells (Figure 3D; Student´s *t*-test *p* < 0.001). Treatment with the different CoQs in the absence of Aβ_25–35_ did not induce significant changes in MMP. However, only CoQ10 could prevent the Aβ_25–35_-induced decrease in MMP compared to the control (Student´s *t*-test and Dunnett´s multiple comparison test, Figure 3D).

### 3.4. The CoQ Isoprenoid Chain Length Determines the Protective Effect against Aβ_25–35_-Mediated Mitochondrial Bioenergetics Alterations

To further investigate mitochondrial damage, we evaluated two priority energetic pathways in cells through the oxygen consumption ratio (OCR) and extracellular acidification rate (ECAR) using Seahorse XFp > technology, following previously standardized protocols. Mitochondrial respiration was quantified by OCR under basal and energy demand conditions (stressed phenotype).

**Figure 4:**
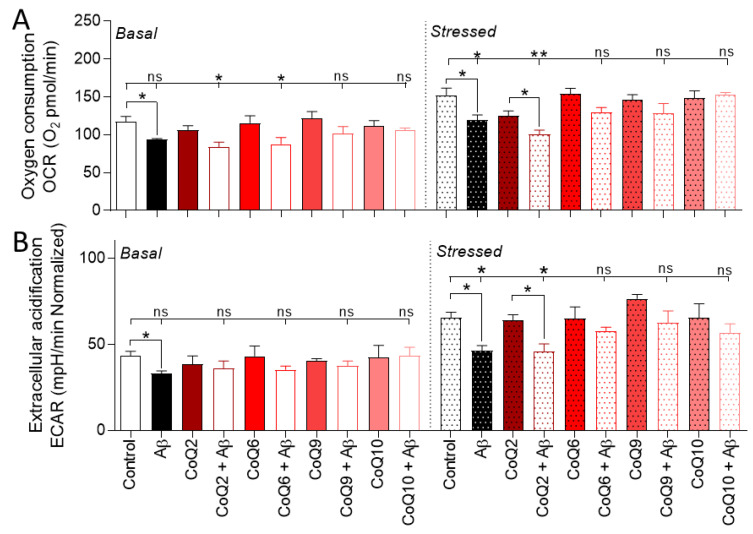
Evaluation of the energetic cellular phenotype in cells exposed to Aβ_25–35_ and different CoQs. bEnd.3 cells were pretreated for 24 h with CoQ2, CoQ6, CoQ9, or CoQ10 and then treated with Aβ_25–35_ for 24 h. (**A**) OCR levels are expressed as oxygen consumption (picomoles) per minute. (**B**) ECAR levels are expressed as changes in pH per minute. Results were normalized against the total number of cells per well. Data are presented as the mean ± SEM. Student’s *t*-test was used to compare the differences between groups treated with the same ubiquinone and Dunnett’s multiple comparison test to compare each Aβ_25–35_ (plus or minus ubiquinone) treatment with the control. ns: not significant, * *p* < 0.05, ** *p* < 0.01 (*n* = 3).

Under basal conditions, Aβ_25–35_ induced a significant decrease of 20% in OCR. Aβ_25–35_ did not alter OCR in the presence of the different ubiquinones (CoQ2, CoQ6, CoQ9, and CoQ10, Student’s *t*-test *p* > 0.05; Figure 4A). The oxygen consumption levels were significantly lower in the CoQ2 + Aβ_25–35_ and CoQ6 + Aβ_25–35_ groups than in the control group, whereas CoQ9 and CoQ10 prevented Aβ_25–35_-induced damage (Dunnett’s multiple comparison test, *p* < 0.05; Figure 4A). Under stressed conditions, wherein an energy demand situation exists due to the exposure to oligomycin and FCCP, the Aβ_25–35_ treatment significantly reduced the OCR level versus the control with an intensity similar to that of the basal conditions (22% Figure 4A stressed; Student’s *t*-test *p* < 0.05). A similar decrease was observed in cells pretreated with CoQ2 plus Aβ_25–35_, whereas pretreatment with CoQ6, CoQ9, or CoQ10 prevented Aβ_25–35_-induced damage (Figure 4B).

At the same time, we quantified the glycolytic function through ECAR. Aβ_25–35_-treatment triggered a 25–30% reduction in ECAR, thus suggesting an induction of a more quiescent phenotype in cells (Figure 4B). Aβ_25–35_ treatment significantly reduced the ECAR level versus the control with an intensity similar to OCR (24%, Figure 4B). Still, the different ubiquinones reversed this effect under all conditions, except CoQ2 under stressed conditions (Student’s *t*-test and Dunnett’s multiple comparison test *p* > 0.05, Figure 4B).

To further explore the protective effect of CoQ10, we performed a Seahorse Mito Stress assay to measure mitochondrial function in cells treated with Aβ_25–35_ and/or CoQ10. For this purpose, different compounds were added sequentially to the cells (oligomycin, FCCP, and a mix of rotenone and antimycin A), which allowed the assessment of ATP-linked respiration, maximal respiration, and non-mitochondrial respiration, as well as the calculation of proton leak and spare respiratory capacity (Supplementary Figure S1A). Basal respiration results obtained were in line with those previously shown (Supplementary Figure S1B). Aβ_25–35_ treatment significantly reduced ATP-linked respiration, maximal respiration, and non-mitochondrial respiration by 17.6%, 15%, and 18%, respectively. Pretreatment with CoQ10 prevented this increase, showing similar levels to control cells (Supplementary Figure S1B). No changes in spare capacity or proton leaking were observed (Supplementary Figure S1B).

### 3.5. CoQ10 but Not Other Ubiquinones Delayed and Decreased β-amyloid Incorporation into Mitochondria in Endothelial Cells

**Figure 5:**
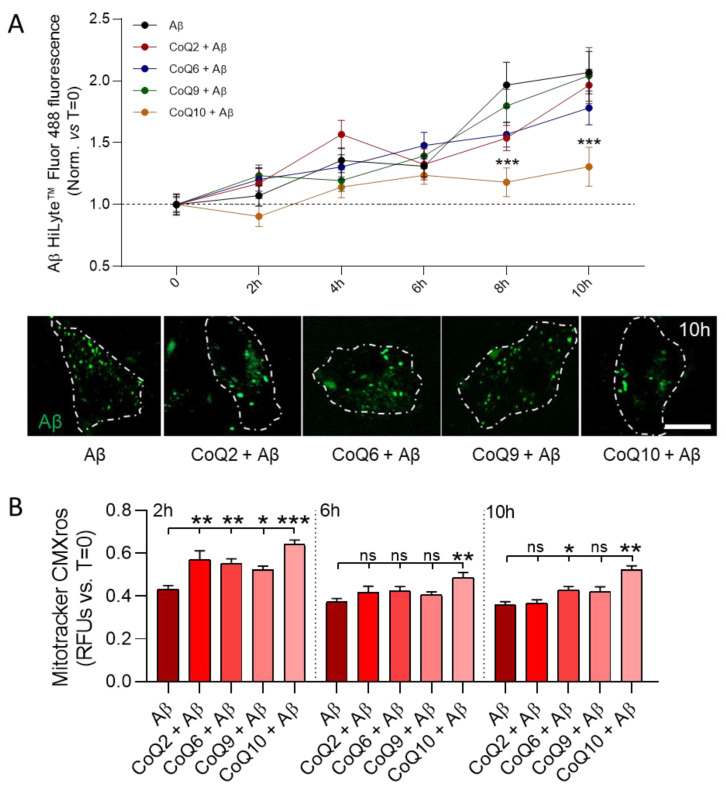
Real-time characterization of Aβ_25–35_ internalization and mitochondrial dynamics. bEnd.3 cells were pretreated with CoQ2, CoQ6, CoQ9, or CoQ10 for 24 h and were then treated with Aβ_25–35_ HyLite^TM^ 488. (**A**) Levels of fluorescent Aβ_25–35_ were measured every two hours and normalized vs. 0 h of exposure. Images show internalized Aβ_25–35_ after 10 h of exposure in individual cells (white line represents the cell outline) (scale bar = 20 µM). (**B**) Mitochondrial status was quantified as the intensity of Mitotracker CMXRos compared to 0 h of exposure to Aβ_25–35_. Data are presented as the mean ± SEM. Dunnett’s multiple comparison test compared each Aβ_25–35_ plus ubiquinone treatment with Aβ_25–35_. ns: not significant, * *p* < 0.05, ** *p* < 0.01, *** *p* < 0.001, (>30 cells/condition).

We next tested the effect of CoQ on the incorporation of the fluorescent Aβ_25–35_ peptide into bEnd.3 cells and, specifically, its accumulation into mitochondria, using confocal microscopy on living cells. The increase in the fluorescent Aβ_25–35_ signal was observed 2 h after peptide administration, and the intensity of the signal progressively increased during the next 10 h of incubation (Figure 5A). Preincubation of bEnd.3 cells with 5 µM CoQ2, CoQ6, and CoQ9 did not alter the amount of fluorescent Aβ_25–35_ detected in the cells.

However, CoQ10 pretreatment resulted in delayed internalization, which was observed after 6 h of Aβ_25–35_ administration and was sustained until the end of the experiment. This significant reduction in uptake reached a decrease of 63.7% compared with control cells after 10 h of incubation (Dunnett’s multiple comparison test *p* < 0.001, Figure 5A, orange line). Mitochondrial damage was evaluated parallel at 2 h, 6 h, and 10 h after Aβ_25–35_ administration.

However, CoQ10 pretreatment resulted in a delayed internalization, which was observed after 6 h of Aβ_25–35_ administration and was sustained until the end of the experiment. This significant reduction in uptake reached a decrease of 63.7% compared with control cells after 10 h of incubation (Dunnett’s multiple comparison test *p* < 0.001, Figure 5A, orange line). Mitochondrial damage was evaluated in parallel at 2 h, 6 h, and 10 h after Aβ_25–35_ administration.

We observed that, after 2 h, the Mitotracker CMXRos signal was higher in cells pretreated with the different CoQs compared to Aβ_25–35_ treated cells (Dunnett’s multiple comparison test *p* < 0.01; Figure 5B), indicating that mitochondrial damage was one of the first cellular alterations induced by Aβ_25–35_. After 6 h and 10 h of incubation, no differences were observed in cells pretreated with CoQ2 and CoQ9 compared to non-pretreated cells, thus indicating that their preventive effect observed at 2 h was not maintained for longer durations. Nonetheless, CoQ10-pretreated cells preserved the mitochondrial staining and, therefore, the MMP, suggesting that CoQ10 protects against Aβ_25–35_-induced mitochondrial dysfunction. It is interesting to note that, although no differences were found for CoQ6 at 6 h, it also showed a protective effect after 10 h of incubation. (Figure 5B).

**Figure 6:**
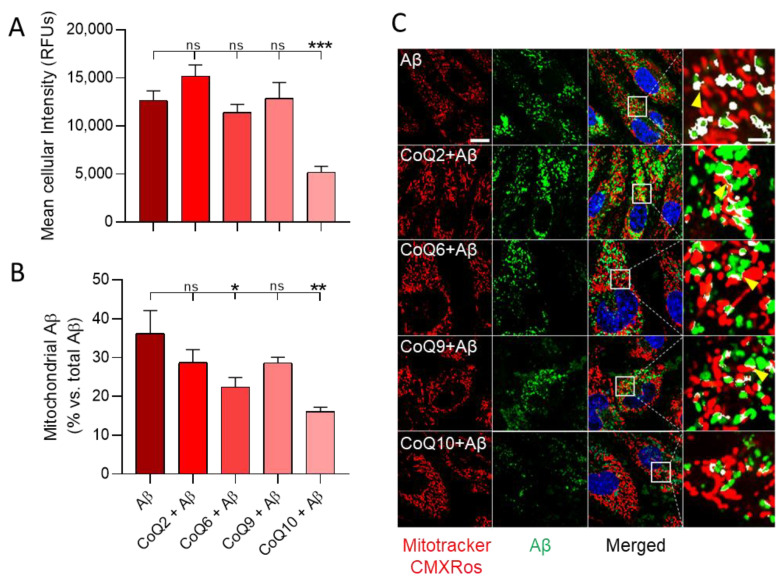
Aβ_25–35_ internalization and mitochondrial colocalization in bEnd.3 cells exposed to Aβ_25–35_ and different CoQs. bEnd.3 cells were pretreated with CoQ2, CoQ6, CoQ9, or CoQ10 for 24 h and then treated with Aβ_25–35_ HyLite^TM^ 488 for 24 h. (**A**) Internalized fluorescent Aβ_25–35_ is expressed as RFUs. (**B**) Mitochondrial-fluorescent Aβ_25–35_ was measured as the percentage of fluorescent Aβ_25–35_ present into mitochondria compared to total internalized Aβ_25–35_. (**C**) Images show cells stained with Mitotracker CMXros (red) and Aβ_25–35_ (green) and their colocalization. Detailed images show the colocalized areas in white (scale = 20 µM/scale augmented = 5 µM). Data are presented as the mean ± SEM. Dunnett’s multiple comparison test compared each Aβ_25–35_ plus ubiquinone treatment with Aβ_25–35_. ns: not significant, * *p* < 0.05, ** *p* < 0.01, *** *p* < 0.001, (*n* = 3).

After 24 h of incubation, whereas Aβ_25–35_ uptake in cells pretreated with CoQ2, CoQ6, and CoQ9 was similar to that of non-pretreated cells, CoQ10-pretreated cells showed a significant reduction of 53% compared to Aβ_25–35_ treated cells (Dunnett´s multiple comparison test *p* < 0.001; Figure 6A). CoQ10 also significantly reduced the internalization of Aβ_25–35_ compared to the other CoQ homologues (Figure 6A). The z-stack projections confirmed that fluorescent Aβ_25–35_ was observed inside the cells (Supplementary Figure S2).

Co-staining with MitoTracker CMXRos revealed the mitochondrial accumulation of fluorescent Aβ_25–35_ (Figure 6B,C and Supplementary Figure S3). After 24 h of incubation, approximately 35% of the fluorescent Aβ_25–35_ incorporated into Aβ_25–35_ treated cells was found in mitochondria. Although CoQ2 and CoQ9 reduced Aβ_25–35_ mitochondrial incorporation (28% of total internalized fluorescent Aβ_25–35_ reached mitochondria), the decrease was not significant. On the other hand, preincubation with CoQ6 and CoQ10 significantly reduced Aβ_25–35_ incorporation into mitochondria (22.4%, *p* < 0.05 and 16.1%, *p* < 0.01, respectively, of total internalized fluorescent Aβ_25–35_ reached the mitochondria). The detailed images also show how Aβ_25–35_ surrounds the mitochondria, implying that the interaction could occur at a mitochondrial membrane level (Figure 6C, yellow arrows). Taken together, these results demonstrate that CoQ10 is the ubiquinone homologue that more efficiently delays and reduces both the uptake of Aβ_25–35_ into bEnd.3 cells and their trafficking and incorporation into mitochondria.

## 4. Discussion

The deleterious effect of soluble Aβ_25–35_ in HUVEC cells involves its incorporation into the membranes, uptake, and trafficking to the mitochondria, resulting in excess free radicals and deregulation of calcium homeostasis, thus leading to the induction of cell death through apoptosis and necrosis. We previously demonstrated that CoQ10 exhibits a cytoprotective role, and it should be administered before endothelial damage is irreversible [17]. In the present study, we first demonstrate that bEnd.3 cells, an endothelial brain-derived cell line, responds similarly to Aβ_25–35_. Additionally, we show that CoQ10, but no other ubiquinone homologues, protects these cells from the Aβ_25–35_ cytotoxic effect.

NADPH oxidase exerts a crucial role in the brain due to its involvement in the generation of oxidative stress triggered by Aβ-toxicity in endothelial, glial cells, and neurons, which constitutes the cause of neuronal death [26,27]. NADPH oxidase is the major source of free radicals in blood vessels and is responsible for the cerebrovascular dysregulation induced by Aβ [28,29]. NADPH oxidase activation induces ROS production in endothelial cells and stimulates downstream signaling pathways, such as ERK1/2 and p38 MAPK, which ultimately trigger neurovascular dysfunction and neuronal death [8,10,11,16,29,30]. This effect can be attenuated in the presence of NADPH oxidase inhibitors or by adding antioxidants and ROS scavengers [26,30,31,32]. Aβ_25–35_ stimulated NADPH oxidase activity in bEnd.3 cells by 40%. A variety of stimuli, such as angiotensin II, oxLDL, high glucose, high salt, or Aβ, have been shown to activate NADPH oxidase, and it has been previously demonstrated that CoQ10 inhibited the activation of NADPH oxidase induced by most of them [8,11,27,29,33]. CoQ10 prevented oxLDL- and angiotensin II-induced up-regulation of p22^phox^ and Nox2 and impaired the assembly of NADPH oxidase in HUVEC cells [8,11]. In the present study, we report for the first time that CoQ10, but no other ubiquinone homologue, inhibits Aβ_25–35_-induced activation of NADPH oxidase in endothelial bEnd.3 cells, thereby reverting the overproduction of ROS and mitochondrial calcium dysregulation, leading to necrosis and apoptosis [17]. Although in this study we did not quantify the expression of the subunits, NADPH oxidase activity was associated with the upregulation of p22^phox^ mRNA [31]. NADPH oxidase is a plasma membrane enzyme, and its activation in endothelial cells involves the translocation of cytosolic oxidases to the plasma membrane and the phosphorylation of p47phox and Rac [32]. This activation induced by angiotensin II is strongly inhibited by CoQ10 [11]. Exogenous CoQ accumulates in the mitochondria, but significant amounts are also incorporated as a very light endo-lysosomal fraction in associated mitochondrial membranes, and endoplasmic reticulum and further in the plasma membrane [33]. Thus, the CoQ10 action on the Aβ-induced increase in the NADPH oxidase activity can be a cytosolic effect.

Remarkably, the other CoQ homologues, especially CoQ9, whose side chain contains only one less isoprenoid and constitutes the predominant form in mouse cells, could not prevent Aβ_25–35_-induced NADPH oxidase activation or reduce Aβ_25–35_-induced apoptosis and necrosis. Nonetheless, they partially reduced ROS levels, exhibiting the antioxidant activity of the different CoQ homologues, though the most efficient in preventing the Aβ_25–35_-induced excess of ROS was CoQ10. Due to its deeper location in the lipid bilayer, CoQ10 has been proposed to be a better antioxidant than other CoQs homologues since this distribution reduces autoxidation of ubisemiquinone and, thereby, ROS generation [34]. The higher activation of NADPH oxidase might also contribute to the lower reduction in ROS levels found with the other CoQ homologues. Furthermore, the extracellular addition of CoQ considerably increased its concentration in the different cell compartments, mainly in mitochondria [33]. CoQ homologue uptake by cells seems to be independent of the side-chain length [33,35,36]. Thus, modifying the CoQ9/CoQ10 ratio naturally present in the cell may explain why some homologues are less efficient in reducing Aβ_25–35_-induced generation of ROS since at considerably higher concentrations than the physiological level, cardiac submitochondrial particles reconstituted with CoQ9 exhibited higher rates of O_2_^−^ generation than those obtained with CoQ10 [34]. Similarly, human cells supplemented with CoQ6 underwent a mitochondrial impairment, not observed with CoQ10 supplementation [35].

The mitochondria is a target for NADPH oxidase-generated ROS, which can induce the opening of the mitochondrial permeability transition pore (mPTP), which causes MMP collapse and ultimately apoptosis [37]. The Aβ_25–35_-induced increase in cytosolic free calcium was also impaired by most of the ubiquinones, but only CoQ10 could prevent the loss of MMP, which would further impair mitochondrial function and apoptosis.

CoQ2, CoQ6, and CoQ10 could only prevent the MMP loss after two hours of Aβ_25–35_ administration, thus suggesting an initial protective effect that cannot be maintained over time. The mPTP presents a ubiquinone-binding site that regulates its aperture, and the interaction of quinones induces mPTP conformational changes that are ligand-specific [38]. Different quinone structural features are required for binding and for stabilizing the pore in the closed conformation. It has been reported that, whereas CoQ5 was ineffective, CoQ10 inhibited its opening and, therefore, prevented apoptosis [38,39]. The effect of CoQ10 in inhibiting mitochondrial depolarization is independent of its antioxidant capacity [39]. Indeed, extracellular incubation with CoQ10 increases its concentration in all cell membranes, and we have described that increasing CoQ10 plasma membrane levels in vivo by dietary supplementation entirely abolishes the aging-related increase in liver plasma membrane neutral sphingomyelinase activity, thereby inhibiting the apoptotic pathway [40].

The mitochondrial damage manifested in this study also entails decreased oxygen consumption, as shown by Seahorse XFp > technology, which allows a real-time and highly accurate assessment of metabolic alterations in cells. This harmful phenotype generated by Aβ_25–35_ was entirely prevented by pretreatment with CoQ10 and partially rescued by other shorter chain CoQs. To date, the effect of Aβ on OCR and ECAR is not entirely clear. A recent study reported that exposure of Aβ for 7 h induces an increase in OCR, accompanied by mitochondrial damage, increased ROS, and electron transport disruption in cerebrovascular endothelial cells [41]. However, in the present study, we observed a decrease in OCR and ECAR induced by Aβ_25–35._ bEnd. 3 cells may show a biphasic response of mitochondrial oxygen consumption upon Aβ_25–35_ administration by exhibiting increased oxygen consumption after short exposure times and a reduction upon more prolonged exposure (24 h), as obtained in our study and previous others [42,43,44,45,46]. This decrease correlates with the alteration of MMP and the increase in the ROS level.

It has been previously shown how the direct and indirect (through ROS increase) interaction of Aβ with mitochondria disrupts the function of different components of the electron transport chain, thus affecting cellular respiration [47,48]. Here, we report that Aβ_25__–__35_ induces an alteration of the whole mitochondrial respiratory chain, decreasing basal respiration, ATP production, and maximal respiration. These effects were prevented by the pretreatment with CoQ10, confirming the results reported by Sadli et al., in neuroblastoma cells [45]. The protective effect of CoQ10 on different respiration parameters confirmed the role of CoQ10 at the mitochondrial level. Pretreatment with CoQ10 also prevented the deleterious effect of Aβ_25__–__35_ on non-mitochondrial respiration (enzymatic oxygen consumption), thus implying a dual effect at both mitochondrial and cytosolic levels. Pretreatment with CoQ9 and CoQ6 prevented the decrease in OCR and ECAR levels generated by Aβ_25–35_ but did not hamper MMP alteration. This may be explained since exposure to these CoQs slightly reduced the interaction of Aβ_25–35_ with mitochondria, but not as markedly as CoQ10, a potent inhibitor of mPTP opening.

Aβ is a highly hydrophobic peptide capable of channel formation in lipid bilayers, a property that has been proposed as part of their pathogenic mechanism because it disrupts intracellular calcium regulation and MMP [49]. We previously reported that CoQ10 protection from Aβ-induced deleterious effects in endothelial cells also involves these mechanisms, reducing Aβ trafficking and accumulation into the mitochondria, impeding both mPTP opening and cytochrome *c* release [17]. In the present work, we describe that only CoQ10 among the ubiquinone homologues tested could reduce the uptake of Aβ_25–35_ into both the cell and the mitochondria and that this effect correlates with the maintenance of MMP. To form a pore into the membrane, Aβ aggregates spontaneously into extended fibrils and precipitates out of solution, and then, dozens of these peptides are inserted in lipid bilayers forming a channel [49]. It has been reported that CoQ10 inhibits the formation of Aβ fibrils and their extension, and it destabilizes preformed fibrillar Aβ [50]. To our knowledge, there are no studies that address the interaction of Aβ with other CoQ homologues. CoQ10 addition to the culture medium of the cells increases the cellular concentration of CoQ10 more than 1000 times compared to the control [36], so one possible mechanism by which CoQ10 decreases Aβ incorporation may be by reducing its membrane insertion or by destabilizing the fibril formed during its insertion into cell membranes. It has been proposed that decreased lipophilicity might reduce Aβ and/or fibrillar Aβ binding affinity and exhibits anti-amyloidogenic and fibril-destabilizing effects in vitro [50]; thereby, the three-dimensional conformation of CoQ10 could be more efficient than other homologues to impede the association between Aβ molecules that leads to fibrillar Aβ formation. Furthermore, the influence of CoQ10 on the permeability and mechanical properties of phospholipid membranes is similar to that of cholesterol [51]. The increase in cholesterol in membranes inhibits Aβ channel insertion in liposomal and cellular membranes, and oppositely, cholesterol depletion facilitates peptide insertion in membranes and pore formation [49].

It is difficult to explain why CoQ10 exhibits a protective effect on endothelial cells from Aβ_25–35_-induced damage while other homologues do not, even though the major form in mouse cells is CoQ9. However, this intriguing difference has been described in other processes such as Mumps virus infection, which causes the death of 30–35% of cultured rat neurons and decreases the concentration of CoQ10 and CoQ9. It was demonstrated that supplementation of the medium with 5 µM CoQ9 did not prevent this neuronal death, whereas CoQ10 supplementation was shown to be protective and increased the number of surviving neurons to 90% in the dorsal root ganglion and cerebral cortex [52]. Additionally, longevity correlates positively with CoQ10 levels and negatively with CoQ9 levels in different mammal species, and it has been suggested that CoQ10 may be a better antioxidant than CoQ9 due to its deeper localization in the lipid bilayer, which reduces autoxidation of ubisemiquinone and thereby ROS generation [34]. We previously demonstrated that CoQ10H_2_ and ascorbate reduce cerebral hypoxia and prevent BACE1 expression and Aβ deposition while reducing the thickness of the cerebrovascular basement membrane during early asymptomatic stages of AD in the 3xTg-AD mice model of AD [18]. Additionally, CoQ10H_2_ reduced chronic inflammation and improved cerebral vasoreactivity and endothelial dysfunction in patients with mild cognitive impairment when the CoQ10H_2_ concentration reached a concentration of 5 µg/mL in plasma [19]. AD is a neurodegenerative disease whose main risk factor is age. The CoQ10/CoQ9 ratio declines during aging, and this process appears to be attenuated by calorie restriction, which increases CoQ10 biosynthesis [53]. Calorie restriction also attenuates AD-type brain amyloidosis in the 3xTg-AD mouse model and monkeys by reducing the Aβ content in the brain [54,55]. Another dietary intervention that favors the synthesis of CoQ10 over CoQ9, thus altering the ratio between CoQ homologues, also ameliorated the risk of many age-related disorders [56].

## 5. Conclusions

The results presented in this work demonstrate that only CoQ10 among tested ubiquinones protects endothelial cells from Aβ_25–35_-induced damage. CoQ10 was the only ubiquinone that inhibited Aβ_25–35_-mediated activation of NADPH oxidase, maintained MMP, and reduced Aβ_25–35_ cellular and mitochondrial uptake, thus suggesting that this protective effect is not only mediated by its antioxidant capacity.

## Data Availability

The data generated during this study are included in this article and supplementary.

## Supplementary Materials

The following are available online at http://www.mdpi.com/s1. Figure S1: Evaluation of cellular mitochondrial respiration in cells exposed to Aβ_25–35_ and different CoQ10, Figure S2. Z-stack projections of cells exposed to Aβ_25–35_ and different CoQs. bEnd.3 cells were pretreated with CoQ2, CoQ6, CoQ9 or CoQ10 for 24 h and then treated with Aβ_25–35_ HyLite^TM^ 488 for 24 h. Images show the z-stack projection of cells stained with Mitotracker (red), Hoechst (blue) and Aβ_25–35_ (green), Figure S3. Representative Confocal images of control cells. Images show cells stained with Mitotracker CMXRos (red) and Aβ_25–35_ (green) and its colocalization. (Scale = 20 µM // Scale augmented = 5 µM).
