# Supplementary material for: The Protective Effect of Ubiquinone against the Amyloid Peptide in Endothelial Cells Is Isoprenoid Chain Length-Dependent
Source: Antioxidants (Basel). 2021 Nov 13;10(11):1806. doi: 10.3390/antiox10111806 (PMC8615161; doi:10.3390/antiox10111806)
Supplement: Supplementary file 1 [file antioxidants-10-01806-s001.zip › antioxidants-1448846-supplementary.pdf]

Supporting Information

The protective effect of ubiquinone against the amyloid peptide in endothelial cells is isoprenoid chain length-dependent

Javier Frontiñán-Rubio [1-2], Yoana Rabanal-Ruiz[1-2], Mario Durán-Prado [1-2*] and Francisco Javier Alcain [1-2,*]

[1] Department of Medical Sciences, Faculty of Medicine, University of Castilla-La Mancha, 13071 Ciudad Real, Spain.
[2] Oxidative Stress and Neurodegeneration Group, Regional Centre for Biomedical Research, University of Castilla-la Mancha, 13071 Ciudad Real, Spain.
* Correspondence: franciscoj.alcain@uclm.es (F.J.A.); mario.duran@uclm.es (M.D.-P.).

Supporting Figure S1

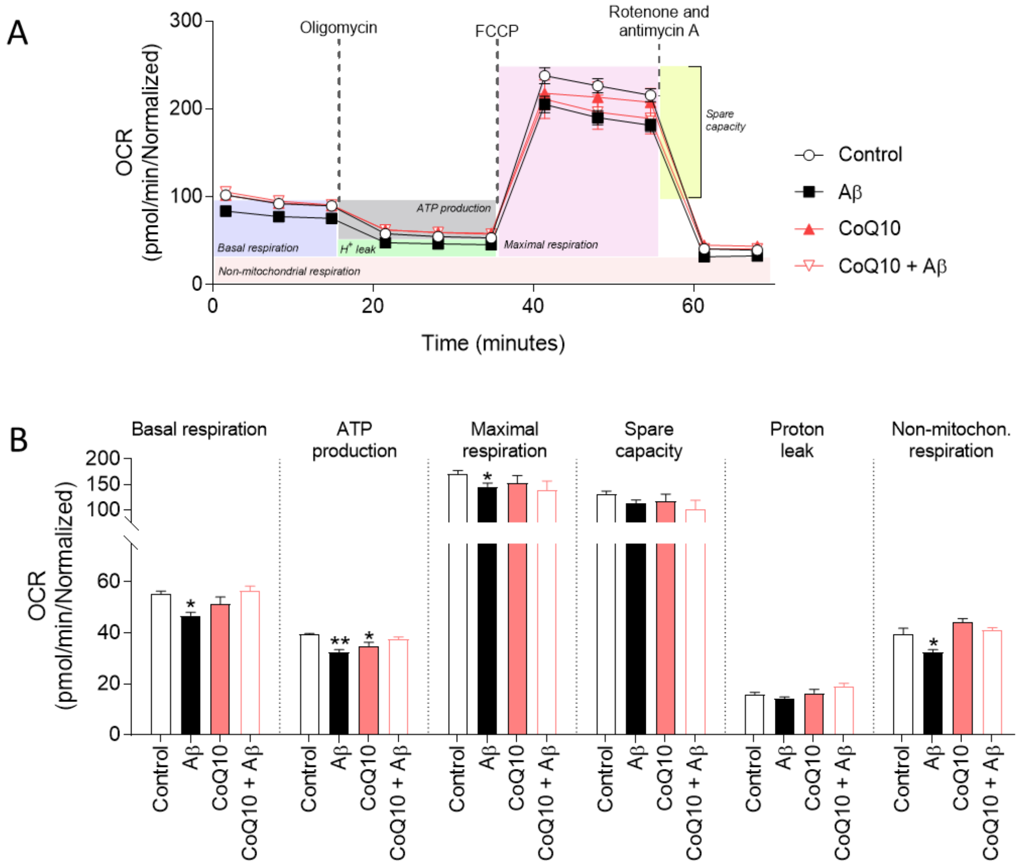

Supp. Figure S1. Evaluation of cellular mitochondrial respiration in cells exposed to $A\beta_{25\text{–}35}$ and different CoQ10. bEnd.3 cells were pretreated 24h with CoQ10 and then treated with $A\beta_{25\text{-}35}$ for 24h. (A) Oligomycin, FCCP, and a mix of rotenone and antimycin A were sequentially injected to measure ATP-associated respiration, maximal respiration, and non-mitochondrial respiration, respectively. Proton leak and spare respiratory capacity were calculated using these parameters and basal respiration. (B) OCR levels are expressed as oxygen consumption (picomoles) per minute. Results were normalized against the total number of cells per well. Data are presented as mean ± SEM. Student's t test was used to compare the differences between groups treated with the same ubiquinone. *p < 0.05, **p < 0.01 (n = 3).

Supporting Figure S2

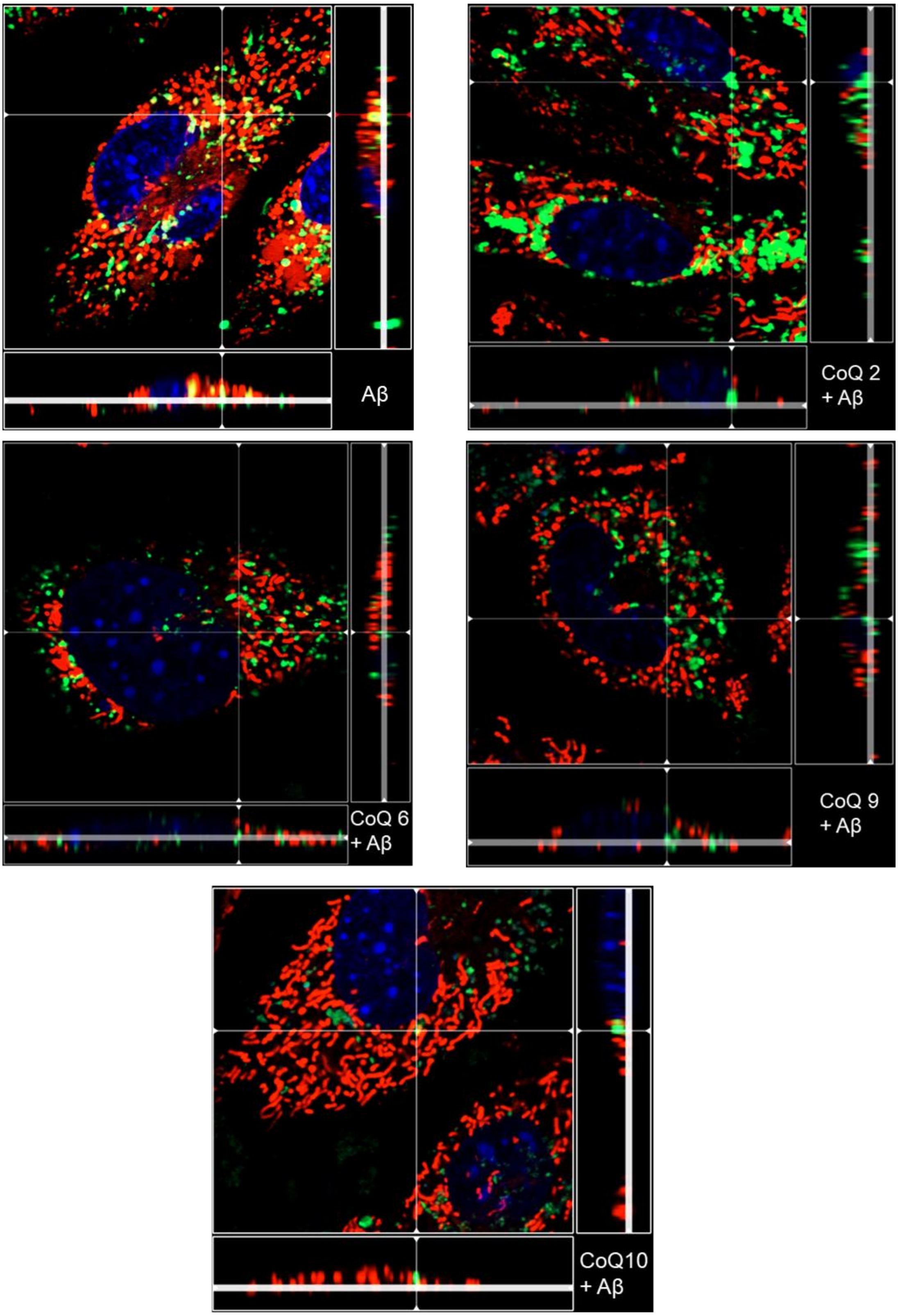

Supp. Figure S2. Z-stack projections of cells exposed to $A\beta_{25\text{–}35}$ and different CoQs. bEnd.3 cells were pretreated with CoQ2, CoQ6, CoQ9 or CoQ10 for 24h and then treated with $A\beta_{25\text{-}35}$ HyLite™ 488 for 24h. Images show the z-stack projection of cells stained with Mitotracker (red), Hoechst (blue) and $A\beta_{25\text{-}35}$ (green).

Supporting Figure S3

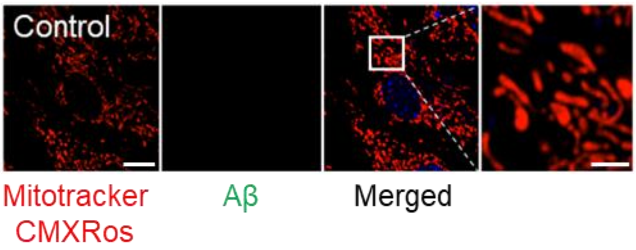

Supp. Figure S3. Representative Confocal images of control cells. Images show cells stained with Mitotracker CMXRos (red) and A$\beta_{25\text{-}35}$ (green) and its colocalization. (Scale=20μM // Scale augmented=5μM).
